# Cellulose Isolation Methodology for NMR Analysis of Cellulose Ultrastructure

**DOI:** 10.3390/ma4111985

**Published:** 2011-11-07

**Authors:** Marcus B. Foston, Chistopher A. Hubbell, Art J. Ragauskas

**Affiliations:** School of Chemistry and Biochemistry, Institute of Paper Science and Technology, Georgia Institute of Technology, 500 10th St., Atlanta, GA 30332, USA; E-Mails: marcus.foston@ipst.gatech.edu (M.B.F.); chis.hubbell@comcast.net (C.A.H.)

**Keywords:** acid hydrolysis, cellulose, crystallinity, isolation method, solid-state NMR, ^13^C CP/MAS

## Abstract

In order to obtain accurate information about the ultrastructure of cellulose from native biomass by ^13^C cross polarization magic angle spinning (CP/MAS) NMR spectroscopy the cellulose component must be isolated due to overlapping resonances from both lignin and hemicellulose. Typically, cellulose isolation has been achieved via holocellulose pulping to remove lignin followed by an acid hydrolysis procedure to remove the hemicellulose components. Using ^13^C CP/MAS NMR and non-linear line-fitting of the cellulose C_4_ region, it was observed that the standard acid hydrolysis procedure caused an apparent increase in crystallinity of ~10% or less on the cellulose isolated from *Populus* holocellulose. We have examined the effect of the cellulose isolation method, particularly the acid treatment time for hemicellulose removal, on cellulose ultrastructural characteristics by studying these effects on cotton, microcrystalline cellulose (MCC) and holocellulose pulped *Populus*. ^13^C CP/MAS NMR of MCC indicated that holocellulose pulping and acid hydrolysis has little effect on the crystalline ultrastructural components of cellulose. Although any chemical method to isolate cellulose from native biomass will invariably alter substrate characteristics, especially those related to regions accessible to solvents, we found those changes to be minimal and consistent in samples of typical crystallinity and lignin/hemicellulose content. Based on the rate of the hemicellulose removal, as determined by HPLC-carbohydrate analysis and magnitude of cellulose ultrastructural alteration, the most suitable cellulose isolation methodology utilizes a treatment of 2.5 M HCl at 100 °C for a standard residence time between 1.5 and 4 h. However, for the most accurate crystallinity results this residence time should be determined empirically for a particular sample.

## 1. Introduction

Bioethanol has been recognized as a potential source to replace current non-renewable resources via biochemical conversion from cellulose [1]. Lignocellulosics are the most abundant terrestrial organic materials, serving as a structural biopolymer in the cell walls of plants. The very properties that make lignocellulose so useful in nature, biomass recalcitrance, also makes it difficult and expensive deconstruct, especially biologically [2,3]. In order to meet global energy demands and produce sufficient biofuel volumes at lower cost, fundamental research has begun to focus on the chemistry, dynamics and mechanism of biomass recalcitrance and mitigation technologies [1,4].

Understanding the origins of biomass recalcitrance and the structural changes that occur during biosynthesis and deconstruction are vital for improving current processing and conversion methods for cellulosic biofuels. For example, efficient enzymatic hydrolysis of lignocellulose has been directly related to cellulase enzyme activity and the potential effect of substrate characteristics, such as crystallinity, degree of polymerization, specific surface area, lignin and hemicellulose distribution [5]. Researchers have employed plant genetic manipulation and thermochemical pretreatments to modify these substrate characteristics, reduce biomass recalcitrance and increase sugars yields from subsequent enzymatic deconstruction [6]. Unfortunately, these methods not only target plant cell wall characteristics of interest but also invariably change a variety of other substrate properties. In particular, hydrothermal pretreatments have been shown to increase enzymatic sugar yields, which have been subsequently correlated to changes in plant cell wall characteristics such as hemicellulose and lignin removal, increases in cellulose accessibility and biomass pore size and decreases in cellulose molecular weight [7,8,9,10,11,12,13]. Many of these same studies also indicate the crystallinity index of cellulose increases with hydrothermal pretreatment, however several studies have reported that a decrease in crystallinity may accelerate enzymatic hydrolysis [9,14,15], demonstrating the rate of enzymatic hydrolysis is much faster with amorphous cellulose. However, the literature does not seem to present a consensus and along with recent work indicating accessibility maybe be one of the major first order rate determining factors [16], observed correlations between crystallinity and hydrolysis yield and rate does not indicate direction causation. This confusion can be contributed to the complex nature of the cell wall and again the fact that deconvolution of substrate properties to enzymatic hydrolysis requires capturing a comprehensive representation of the cell wall on multiple length scale, which few laboratories have the capabilities to accomplish. This suggests cellulose ultrastructure, though not as critical as once thought, is factor in enzymatic deconstruction of biomass which must be monitored if only to remove or confirm its minor affect on recalcitrance. Therefore, having techniques to accurately probe and track cellulose ultrastructure are critical to deconvoluting recalcitrant relevant plant cell wall characteristics and future improvements in the production of biofuels.

Infrared-red (IR) and Raman spectroscopy, along with X-ray diffraction (XRD) and electron diffraction/microscopic methodologies have been used to analyze cellulose ultrastructure; however no technique alone can fully characterize the ultrastructure of cellulose with respect to the proportions and exact nature of amorphous, crystalline and p*ara*-crystalline cellulose [17,18]. Another useful technique to analyze chemical and ultrastructural features of cellulose is nuclear magnetic resonance (NMR). NMR is sensitive to the magnetic non-equivalences in an environment of chemically equivalent nuclei. Consequently, ^13^C cross polarization magic angle spinning (CP/MAS) NMR spectroscopy has been used to analyze the ultrastructure of cellulose for more than thee decades [15,18,19,20,21,22,23,24,25,26,27,28,29,30,31,32,33,34,35,36,37,38,39,40]. One of the first applications of ^13^C CP/MAS NMR to investigate the ultrastructure of native cellulose was reported by Atalla and Vanderhart [18,19,20,26,27]. These studies employing cellulose from *Acetobacter, Valonia*, Kraft pulp and low-DP acid-hydrolyzed cellulose reported unique chemical shifts for the C_4_ and C_6_ carbon positions in the anhydroglucose unit dependent upon the cellulosic source. They proposed this was due to the rigid and highly hydrogen-bonded cellulose crystalline structure, in which the highly varied ultrastructure of cellulose, related to the cellulosic source, generated magnetic non-equivalences [18]. These non-equivalences were reflected in the spectra as variations in C_4_ and C_6_ carbon chemical shifts and related to the ultrastructure of cellulose. 

The C_1_, C_4_ and C_6_ signals of cellulose extend over chemical shift ranges of δ ~102–108, 80–92 and 57–67 ppm, respectively and display the best ^13^C chemical shift dispersion of carbons in the anhydroglucose unit. Of those three regions, the C_4_ peak is most commonly used, and probes the cellulose amorphous domain which appears as a fairly broad signal from δ ~80–85 ppm. The C_4_ crystalline domain produces a sharper resonance from δ ~85–92 ppm [18].

Typically, a two peak non-linear least-squared model or basic peak integration is used to determine the degree of crystallinity based upon the relative area of the amorphous and crystalline resonances in the C_4_ region. Park *et al*. [41] has reported a comprehensive comparison of degree of crystallinity as determined by solid state ^13^C NMR utilizing either peak integration or a novel amorphous cellulose NMR spectral subtraction procedure to X-ray diffraction data analyzed by several different methods used to extract crystallinity information from diffraction patterns. This work not only described a new method for processing NMR spectra of cellulose to determine degree of crystallinity but also, and more importantly, demonstrated that NMR spectral fitting can accurately detect relative changes in crystallinity from different cellulose samples.

The NMR spectrum of cellulose, like many biological systems with complex chemical and structural moieties, contains broad and overlapping peaks. Via solvent exchange and ^13^C spin-lattice relaxation experiments literature studies have identified several peak assignments within the spectrum of cellulose attributed to *para*-crystalline cellulose and amorphous accessible or inaccessible fibril surfaces. Resonances originating from *para*-crystalline cellulose and amorphous fibril surfaces displayed distinct T_1_ relaxation rates on an order of magnitude shorter than the resonances attributed to cellulose I_α_ and cellulose I_β_ [24]. Also, accessible fibril surfaces demonstrated resonances displayed chemical shift changes after solvent exchange (not observed for inaccessible fibril surface related resonances) [24]. The exact nature and origins of resonances from p*ara*-crystalline cellulose and cellulose fibril surfaces has been an area of debate though the existence of these individual NMR-signals within the overlapping cellulose spectrum is well established. Work by Ding *et al*. [42] utilizing high-resolution atomic force microscopy on the surface of cellulose microfibrils suggested that the elementary cellulose fibril is composed of 36 glucan chains, where the most inner core six chains are true crystalline chains. Those core chains are surrounded by 12 *para*- or sub-crystalline chains, which are then surrounded by 18 more sub-crystalline or non-crystalline chains. NMR methodologies not only estimate relative amounts of cellulose crystal allomorphs but also deconvolute contributions of *para*-crystalline cellulose, accessible and inaccessible fibril surfaces, which has been refined to include the non-linear line-fitting of six or seven resonances of adjustable shape, width, chemical shift, and relative intensity. 

The determination of cellulose crystallinity and spectral fitting of NMR data to estimate the relative amounts of the various ultrastructural components of cellulose is fairly straightforward on pure samples of cellulose [24,31]. In 1994, Lennholm *et al*. [31] developed a novel method utilizing a partial least-squares (PLS) model, a precursor to the analysis described above, to estimate the relative amounts of cellulose crystallinity index involving cellulose I_α_ and cellulose I_β_-content on variety of calibration samples such as cellulose from *Valonia ventricosa*, bacterial cellulose from *Acetobacter xylinum*, cotton linters, *etc*. However, wood pulp and other biomass samples contain other overlapping NMR-signals from hemicellulose and lignin side-chains that can contribute to the signal intensity of resonances of interest. Earlier work by Newnan *et al*. [39] used spectral editing NMR sequences, specifically a T_1ρ_ spin-lock filter prior to the contact pulse in the CP sequence to probe crystallinity in wood pulp samples [32,40]. There are several other spectral editing techniques based on other T_2_ and T_1_ relaxation phenomena that have been reported [32,39,43]. In general, spectral editing techniques work by allowing most of the NMR-signal of hemicellulose and lignin side-chains to decay or relax while retaining the signal from cellulose due to differences in relaxation rates. Though this technique is particularly useful in qualitative analyses of wood samples, the fact that crystalline and amorphous cellulose have different relaxation rates makes extracting quantitative information difficult.

In an effort to further study the non-crystalline forms within biomass containing cellulose I by ^13^C CP/MAS NMR Larsson *et al*. [22] prepared bleached birch kraft pulp with a method originally cited to prepare hydrocellulose [44] by refluxing in 2.5 M HCl for various residence times. In doing so, they proposed four hours of hydrolysis time was the optimal compromise between removing most of the hemicelluloses with minimal cellulose degradation. Following this study, the standard sample preparation of cellulose from wood for ultrastructural analysis by ^13^C CP/MAS NMR involved removing the lignin component by holocellulose pulping followed by an acid hydrolysis to remove the hemicellulose component. Alkaline extraction of hemicellulose is also very effective at the removal of hemicellulose and may cause less severe degradation of residual cellulose, however Iverson and other researchers have avoided this method due to cellulose mercerization which may convert cellulose I to the cellulose II [24,45,46].

There are other complementary methods, primarily X-ray diffraction and IR spectroscopy, which have been employed to determine cellulose crystallinity in wood fibers [3,47]. However, crystallinity data from these different analytical techniques has been shown to demonstrate dissimilarity in results on the same sample, most likely due to varying principles of measurement. In the aforementioned study by Park *et al*. [41], eight different cellulose preparations produced significantly different crystallinity values depending on the method used. Nevertheless, for each technique tested the resulting crystallinity index of the various cellulose preparations ranked similarly and the relative changes seemed consistent. Moreover, with both techniques, obscuring signals from lignin and hemicelluloses can complicate accurate analysis of the cellulose ultrastructure. This therefore makes cellulose isolation and determining the effect of cellulose isolation on the ultrastructure even more relevant.

In this study, we examine in greater detail ^13^C CP/MAS NMR ultrastructural analysis of cellulose in conjunction with HPLC carbohydrate analysis to assess residual hemicellulose distributions and gel permeation chomatography (GPC) to probe cellulose degradation. All these studies were performed to determine the effect of cellulose isolation methodologies on cellulose and its ultrastructure. Holocellulose pulped *Populus*, cotton and microcrystalline cellulose (MCC) samples were refluxed in 2.5 M HCl for various residence times in an attempt to further define the changes occurring in the ultrastructure of cellulose during these chemical treatments used to isolate cellulose and how that could alter our interpretation of the resulting spectral data. 

## 2. Results and Discussion

To comprehensively study the effect of cellulose isolation method on the ultrastructure of cellulose, we chose to first evaluate the efficiency of hemicellulose removal via HPLC carbohydrate analysis of a time-series of acid hydrolysis reactions on holocellulose pulped *Populus*. Once a rate on hemicellulose extraction is established, the magnitude of changes in the ultrastructure of cellulose by ^13^C CP/MAS NMR and cellulose degradation rates as acquired by GPC were determined. This was done on a variety of substrates (cotton, microcrystalline cellulose and holocellulose pulped *Populus*) and used to model native cellulose within a cell wall, evaluating changes in the ultrastructure of cellulose during hemicellulose extraction.

### 2.1. Hemicellulose Removal

**Figure 1:**
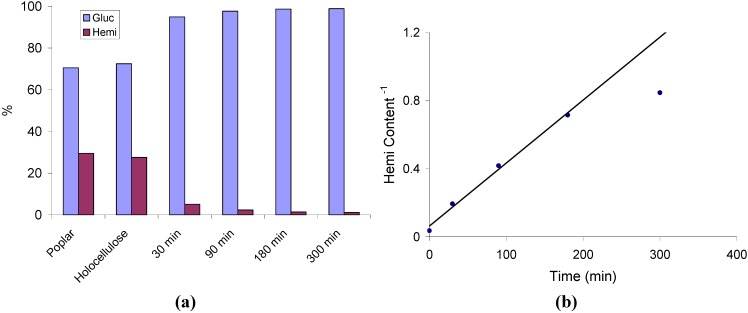
(**a**) Carbohydrate distribution and (**b**) reciprocal of the hemicellulose (Hemi) content for a *Populus* standard after holocellulose pulping and hemicellulose removal by acid hydrolysis in 2.5 M HCl at various residence times.

To effectively analyze the ultrastructure of cellulose using ^13^C CP/MAS NMR the majority of lignin and hemicellulose, which contain signals in the same spectral region as the C_4_ resonance, must be removed. The removal of hemicellulose relies on an acid hydrolysis procedure that was reported by Larsson *et al*. [22,25,44]. In order to develop a more complete description of the changes occurring to the ultrastructure of cellulose a time-resolved study of the hemicellulose removal procedure from holocellulose pulped *Populus* included refluxing in 2.5 M HCl for varying times up to 300 min. HPLC-based monosaccharide anionic exchange chomatography of the pulp was used to quantitatively analyze the changes in the composition of the residual cell wall carbohydrates. The hemicellulose content, characterized by the xylose, mannose, arabinose, and galactose contents are combined, whereas the cellulose content is represented by the glucose content. Figure 1a summarizes the changes in carbohydrate distribution at various points during the acid hydrolysis. The first data points in Figure 1a indicate that holocellulose pulping removes almost no hemicellulose sugars and that removal of hemicellulose by acid hydrolysis to only be significant after 30 min. The hemicellulose content seemingly plateaus at 1–2% after 90 min of reflux under the reaction conditions employed. 

Plotting the reciprocal of the residual hemicellulose content versus time during acid hydrolysis, as seen in Figure 1b, provides a linear relationship suggesting a second order kinetic for the first ~3 h. Based upon these results, the reaction half-life of hemicellulose removal is ~13.5 min, though this value is not completely accurate as it seems the rate of hemicellulose removal slows with reaction time, deviating from a single second order rate constant near the end of the hydrolysis treatment. Utilizing this rate, alterations within the cellulose ultrastructure and cellulose degradation can be quantitatively compared to hemicellulose removal in a continuous fashion. 

### 2.2. Changes in Ultrastructure and Crystallinity 

In an effort to better understand the ultrastructural changes occurring within cellulose during cellulose isolation our initial studies focused on microcrystalline cellulose (MCC). MCC is prepared by exposing a bleached delignified cellulosic source to a mineral acid solution which removes some of the cellulose amorphous regions and leaves an enriched crystalline microfibril. Its ubiquitous use as model cellulose substrate and the highly crystalline nature of MCC substrate makes it an attractive substrate to analyze potential alterations to the relative proportions of crystalline allomorphs during holocellulose pulping and the acidic hemicellulose extraction treatment.

**Figure 2:**
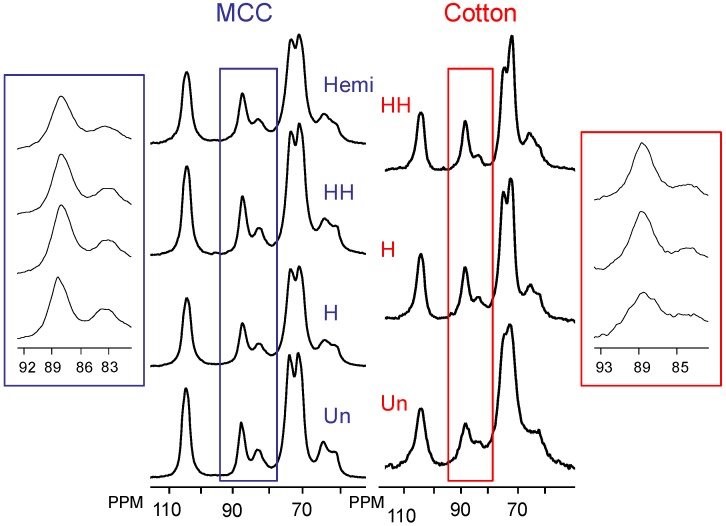
^13^C CP/MAS spectra of microcrystalline cellulose (MCC) and cellulose from cotton swaps after various treatments used to isolate cellulose from whole biomass samples. The ± value represents thee standard deviation of typical variation associated with the measurement. Un = untreated, Hemi = hemicellulose removal by acid hydrolysis, H = holocellulose pulped, HH = holocellulose pulped and hemicellulose removal by acid hydrolysis.

**Figure 3:**
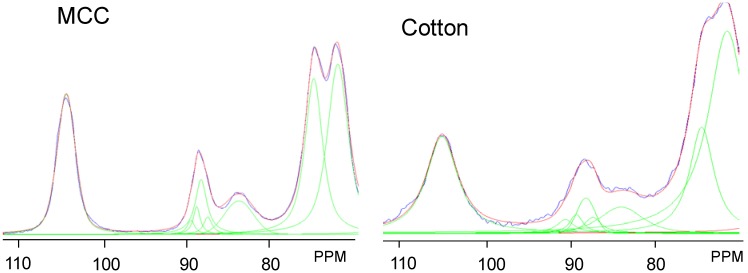
Non-linear line-fits of the ^13^C CP/MAS spectra of untreated microcrystalline cellulose (MCC) and cellulose from cotton swaps.

In order to probe changes occurring in the cellulose crystalline ultrastructure, the relative intensity of the ultrastructural components within cellulose microfibrils and how those relative intensities change with various treatments were evaluated by ^13^C CP/MAS NMR as seen in Figure 2. Crystallinity was determined via 2-peak integration of the C_4_ crystalline carbon region (δ 85–92 ppm) over the integral of the entire C_4_ region (δ 80–92 ppm). Also, ultrastructural components in the crystalline regions of cellulose were derived by line-fitting one Gaussian and thee Lorentzian line-shapes to the C_4_ crystalline carbon signals from δ 85–92 ppm, which are attributed to domains of cellulose I_α+β,_ I_α_, I_β_ and *para*-crystalline cellulose, a procedure described in the literature [24,31] (non-crystalline contributions to crystalline carbon signals were accounted for by the simultaneous mathematical treatment of the δ 80–95 ppm region). The non-linear line-fit of the untreated samples are shown in Figure 3, with the corresponding line parameters in Supplementary-Tables S1 and S2. Lateral fibril dimensions (LFD) can be estimated using the relative intensity of peaks attributed to amorphous cellulose, considered in a square cross-sectional cellulose microfibril model for NMR analysis by Huex *et al*. [48] as total fibril surfaces [43,49]. Based on those intensities and the microfibril model comprised of chains having a width of 0.55 nm, the LFD were also estimated as reference, elucidating the size of the fibril in which the various phases of cellulose I_α_, I_β_ and *para*-crystalline cellulose exist (see Table 1). 

**Table 1:** Crystalline ultrastructural results for line-fitting of ^13^C CP/MAS spectra of microcrystalline cellulose (MCC) and cellulose from cotton swaps after various treatments used to isolate cellulose from whole biomass samples. The ± value represents thee standard deviation of typical variation associated with the measurement. Un = untreated, Hemi = hemicellulose removal by acid hydrolysis, H = holocellulose pulped, HH = holocellulose pulped and hemicellulose removal by acid hydrolysis. The plus-minus values represent thee standard deviations of typical variation associated with the measurement.

Sample	I_α_ (%) ±3	I_α+β_ (%) ±4	Para (%) ±6	I_β_ (%) ±6	CrI (%) ±2	LFD (nm) ±0.5
Un MCC	8	11	35	8	62	5.2
H MCC	7	8	35	10	60	4.9
HH MCC	6	10	42	11	68	6.3
Hemi MCC	7	11	40	19	66	5.9
Un cotton	9	9	35	9	62	5.2
H cotton	6	13	40	6	66	5.9
HH cotton	6	14	49	6	79	9.9

The data in Table 1 represents the results of this fitting procedure on ^13^C spectra of MCC which has undergone the hemicellulose extraction procedure along with other procedures used to isolated cellulose from native biomass. This data suggest little or no change occurs with respect to the crystallinity with holocellulose pulping. More importantly, there seems to only be a slight increase in crystallinity upon treatment under the conditions used for acid hydrolysis of hemicellulose. However, this may be due, in part, to the inherently high proportion of crystalline cellulose which results from the preparation of MCC. Therefore, in an effort to observe the ultrastructural changes occurring within cellulose during cellulose isolation on a substrate with an ultrastructure more comparable to native cellulose a cotton sample was studied using the same protocols used on MCC. As summarized in Table 1, the relative proportions of the cellulose I_α_, I_β_ and *para*-crystalline cellulose were found not to undergo any significant changes when holocellulose pulped. 

The crystallinity and relative proportion of *para*-crystalline cellulose in cellulose from cotton does however appreciably increase upon acid hydrolysis. This change in ultrastructural behavior is indicative of the increased amorphous content in the cotton, suggesting spatially localized hydrolysis preferably degrades amorphous cellulose, increasing the crystallinity. This additional crystallinity is perceived as sub-crystalline cellulose units. This later point will be further discussed with respect to cellulose degradation and is a key point in the overall conclusions of this work. 

**Figure 4:**
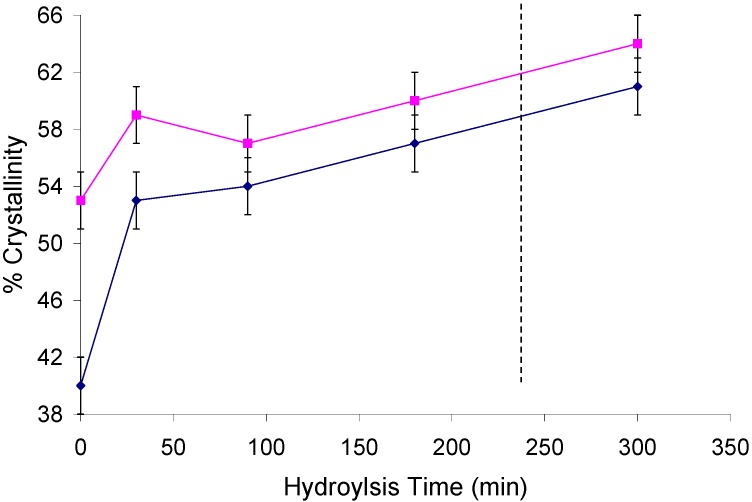
Degree of crystallinity of cellulose from a *Populus* standard after holocellulose pulping and hemicellulose removal by acid hydrolysis in 2.5 M HCl at various residence times as measured by a ^13^C CP sequence (◆) and ^13^C CP sequence with 6.5 ms ^1^H spin-lock filter (■). The dotted line represents the normal 4 h reaction time for hemicellulose removal typically employed. The error bars represent thee standard deviations of typical variation associated with the measurement.

The plot in Figure 4 shows the change in crystallinity with increasing residence time in the hemicellulose removal procedure on holocellulose *Populus*. The C_4_ signals from the cellulose and hemicellulose peaks overlap even in partially acid hydrolyzed samples which can skew the resulting degree of crystallinity determination for cellulose. In an effort to overcome the presence of the hemicellulose resonances, both traditional ^13^C CP experiments and CP experiments with a 6.5 ms ^1^H spin-lock filter (a published and validated procedure to estimate the degree of crystallinity in pulps) were conducted and reported in Figure 4. Hemicellulose ^13^C NMR signals arise at δ 83.5 and 81.7 ppm [50]. In addition, the hemicellulose acetyl groups of *Populus* with resonances at δ ~174 and 21.5 ppm [51] were readily observed. Experimental ^1^H T_1ρ_ values for crystalline and non-crystalline cellulose C_4_ protons in bleached Kraft pulps determined by Newman *et al*. [39], which were similar to values measured in this study, were ~13.6 and 12.7 ms [39], respectively. Whereas the acetate group displayed a much lower ^1^H T_1ρ_ value of ~5.0 ms, [39] this could in part be due to the additional rotational motion of the *O*-methyl bond. However, Newman *et al*. [39] concluded that the acetate groups are so intimately distributed in the hemicellulose that nuclear spin diffusion results in an average T_1ρ_ value. Therefore, the spin-lock filter time was determined by the characteristic ^1^H T_1ρ_ time of the acetate group at δ 21.5 ppm and cellulose C_4_ resonance, allowing a sufficient portion of the hemicellulose magnetization to attenuate during spin-locking before cross-polarization [39]. Both curves display very similar rates of increase with respect to crystallinity and seem to suggest an increase in crystallinity of ~10% or less on cellulose isolated from *Populus* holocellulose after refluxing for 4 h in 2.5 M HCl. Untreated *Populus* standard after holocellulose pulping showed a % crystallinity of 53 and 40 for the normal and spin-lock filtered ^13^C CP sequence, ultimately increasing to 64 and 61% after 5 hours of hemicellulose removal by acid hydrolysis in 2.5 M HCl.

### 2.3. Cellulose Degradation

**Figure 5:**
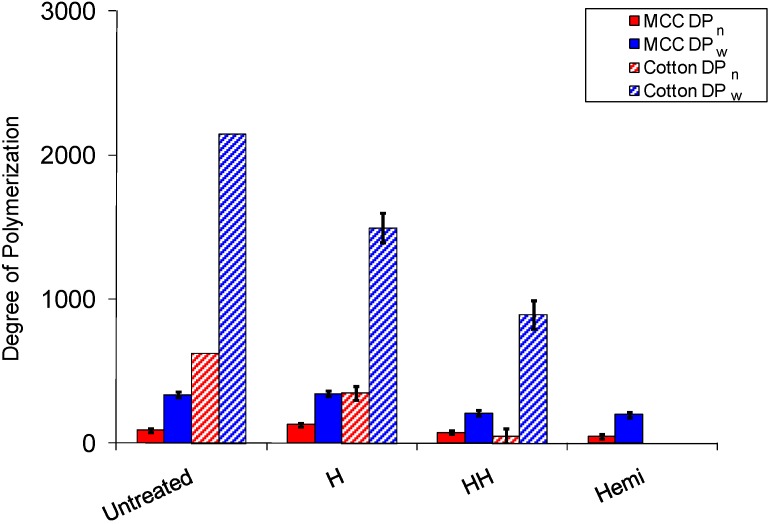
Degree of polymerization of microcrystalline cellulose and cellulose from cotton swaps after various treatments used to isolate cellulose from whole biomass samples. The error bars represent the standard deviations. Hemi = hemicellulose removal by acid hydrolysis, H = holocellulose pulped, HH = holocellulose pulped and hemicellulose removal by acid hydrolysis.

To assess possible degradation and changes in the cellulose ultrastructure due to acid hydrolysis during the aforementioned isolation procedures, a sample of MCC and cotton was holocellulose pulped and refluxed in 2.5 M HCl and the changes in number- and weight-average DP were determined and plotted in Figure 5. There are several methods, including vapor pressure osmometry, reducing end concentration, electron microscopy, light scattering, sedimentation equilibrium, X-ray small angle scattering and intrinsic viscosity, which can be used to determine the relative molecular weight of cellulose [16]. However a commonly utilized technique generates cellulose tricarbanilate with phenyl isocyanate to facilitate dissolution in tetrahydrofuran (THF) and elution in a gel permeation chomatographic (GPC) system. This method requires dissolution by derivatization, specialized chomatography and external standards; however, unlike other direct methods such as the phenol-sulfuric acid method [52] is the only technique which provides a true molecular weight distribution.

As expected, holocellulose pulping conditions had little effect on the MCC molecular weight (see Figure 5). However, significant reductions in DP were observed for cellulose obtained from cotton when subjected to holocellulose pulping. These observations are similar and supported by recent studies by Hubbell *et al*. [53] which showed MCC and cotton linters with ~30% lignin manually incorporated into the samples had only a 1 and 12% reduction in DP, respectively. However, in comparison a 5 and 35% reduction in DP for the ‘lignin-free’ samples were reported upon acid-chlorite holocellulose pulping. In particular, the results on the cotton linters seem to suggest that even though amorphous components of cellulose may display a significant rate of hydrolysis during holocellulose pulping condition when the cellulose is encased in a hemicellulose and lignin matrix the degradation is nominal. When analyzing the effect of holocellulose pulping on cotton in this study, a significant reduction in DP was detected after hemicellulose removal on the substrate, which again would be mitigated by the presence of lignin and hemicellulose in an intact cell wall.

**Figure 6:**
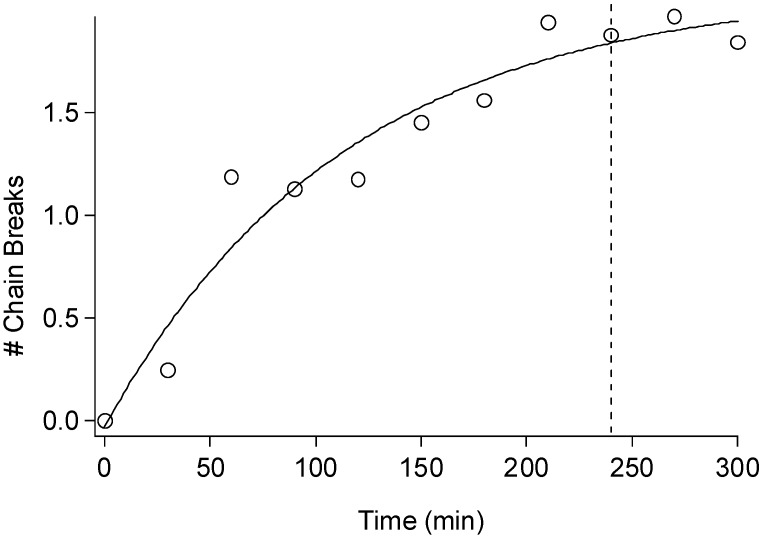
Number of chain breaks of cellulose from a *Populus* standard after holocellulose pulping and hemicellulose removal by acid hydrolysis in 2.5 M HCl at various residence times (# Chain Breaks = (DP_n_^o^/DP_n_) − 1). The dotted line represents the normal 4 h reaction time for hemicellulose removal typically employed and the solid line represents an exponential least-squared fit (# of chain breaks = B + A*exp(time/τ); B = 2.1; A = −2.0; τ = 114.9 min).

A key consideration in determining the proper conditions to remove hemicellulose from biomass is the efficiency of hemicellulose removal versus the degradation of cellulose. GPC characterization of α-cellulose [54] isolated from *Populus* was studied in a similar time-resolved fashion to establish the rate at which cellulose is degraded by the acid hydrolysis. Figure 6 shows a very typical scheme used to represent cellulose degradation, a plot related to number of chain breaks with respect to the initial number-average DP of cellulose as a function of reaction time, where the dotted line represents the 4 h typically associated with the hemicellulose extraction procedure. The results in Figure 4 are similar to a more detailed study by Stephens *et al*. [55] conducted on the hydrolysis of the amorphous cellulose in cotton-based paper. They determined that degradation by acid hydrolysis can be considered as having thee major stages. The initial stage was described as rapid hydrolytic attack on the more solvent accessible amorphous chain segments, while the latter stages display a much slower hydrolytic attack breaking amorphous chain segments on and/or at crystal surfaces. More importantly, the overall degradation process was characterized as exponential having a first-order kinetic. The increase in the number of cellulose chain breaks seen in Figure 4 occurs as an exponential function, suggesting a first order kinetic for the hydrolysis of cellulose during the hemicellulose acid hydrolysis procedure. The average reaction half-life of cellulose degradation during acid hydrolysis in both amorphous and crystalline regions (~79 min) is about 6 times that of hemicellulose removal. Correspondingly, the only large increases in crystallinity occurred in samples which were small molecule extracted, holocellulose pulped and acid hydrolyzed to remove hemicellulose. These same samples consistently were also associated with cellulose degradation equaling # of chain breaks >2. 

Alternative methods of ultrasound- or organic solvent-assisted acid/base extraction of hemicellulose were also attempted in our lab and did not appreciably improve the hemicellulose extraction methodology. The results of this work clearly indicate there is no method to isolate cellulose from lignocellulosic biomass which can leave the cellulose in an unchanged, native state. The use of enzymes to remove both lignin and hemicellulose can be found in a few literature examples, yielding cellulose in almost quantitative yield however the impurities in the cellulose (residual proteins) can be substantial [56]. These impurities can be difficult to remove and make subsequent analytical techniques measuring chemical- or ultrastructure difficult. Furthermore, only about a third of the xylan present in birch pulp was found to be accessible to digestion by xylanases or extraction with 5 wt % potassium hydroxide [50], which was considered dilute enough to not cause mercerization. 

## 3. Experimental Section 

### 3.1. Substrates

*Populus* (*Populus trichocarpa x deltoides*) samples were harvested between 2007–2008 by National Renewable Energy Laboratory (NREL) at area 0800 at Oak Ridge National Laboratory, TN, USA. Microcrystalline cellulose and cotton were purchased from Sigma-Aldrich (St. Louis, MO, USA) and used as received. The biomass was size-reduced in a Wiley mill to a maximum particle size of 0.841 mm, and then fines less than 0.177 mm were removed with a sieve screen. Extractives were subsequently removed by placing the biomass into an extraction thimble in a Soxhlet extraction apparatus. The extraction flask was filled with 1:2 ethanol/toluene mixture (~150 mL) and then refluxed at a rate which cycled the biomass for at least 24 extractions over a 4 h period. The extracted samples were then air-dried prior to further study. 

### 3.2. Carbohydrates and Klason Lignin Analysis

Samples for carbohydrate constituents and acid-insoluble residue (Klason lignin) analysis milled *Populus* were prepared using a two-stage acid hydrolysis protocol based on TAPPI methods T-222 om-88 with a slight modification. The first stage utilizes a severe pH and a low reaction temperature (72 vol.% H_2_SO_4_ at 30 °C for 1 h). The second stage is performed at much lower acid concentration and higher temperature (3 vol. % H_2_SO_4_ at 121 °C for 1 h) in an autoclave. The resulting solution was cooled to room temperature and filtered using G8 glass fiber filter (Fisher Scientific, Hanover Park, IL, USA). The remaining residue which is considered as Klason lignin was oven-dried and weighed to obtain the Klason lignin content. The filtered solution was analyzed for carbohydrate constituents of the hydrolyzed *Populus* samples determined by high-performance anion-exchange chomatography with pulsed amperometric detection (HPAEC-PAD) using Dionex ICS-3000 (Dionex Corp., Sunnyvale, CA, USA) [57]. 

### 3.3. Holocellulose Pulping

Isolated cellulose was generated by first isolating holocellulose from extracted milled biomass pulp. Holocellulose was isolated from extracted samples by exposure to NaClO_2_ (1.30 g/1.00 g lignocellulosic dry solids) in acetic acid (375 mL of 0.14 M) at 70 °C for 2 h. The samples were then collected by filtration and rinsed with an excess of DI filtered water. This was repeated at least once to ensure removal of the majority of the lignin component and then washed with DI water to yield holocellulose pulped samples. Holocellulose samples were shown to contain ~3.7% Klason lignin after pulping twice.

### 3.4. Cellulose Isolation for GPC Analysis

Isolated cellulose was prepared from the holocellulose sample (1.00 g) by extraction with a 17.5% NaOH solution (50 mL) at 25 °C for 30 min. 50 mL of DI filtered water was then added to the NaOH solution. The extraction was continued with the 8.75% NaOH solution (43 mL) at 25 °C for an additional 30 min. Cellulose isolated in this manner is commonly referred to as α-cellulose. The isolated α-cellulose samples were then collected by filtration and rinsed with 50 mL of 1% acetic acid, an excess of DI filtered water, and air dried. 

### 3.5. Gel Permeation Chomatography (GPC) Analysis of Cellulose

The number-average molecular weight (M_n_) and weight-average molecular weight (M_w_) were determined by GPC after tricarbanilation of cellulose [6,54]. Lignin-free cellulose (15 mg) from each sample was placed in separate test tubes equipped with micro stir-bars and dried overnight under vacuum at 40 °C. The test tubes were then capped with rubber septa. Anhydrous pyridine (4.00 mL) and phenyl isocyanate (0.50 mL) were added sequentially via syringe. The test tubes were placed in an oil bath at 70 °C and allowed to stir for 48 h. Methanol (1.00 mL) was added to quench any remaining phenyl isocyanate. The contents of each test tube were then added dropwise to a 7:3 methanol/water mixture (43 mL) to promote precipitation of the derivatized cellulose. The solids were collected by filtration and then washed with methanol/water (1 × 50 mL) followed by water (2 × 50 mL). The derivatized cellulose was then dried overnight under vacuum at 40 °C. Prior to GPC analysis the derivatized cellulose was dissolved in THF (1 mg/mL), filtered though a 0.44 μm filter and placed in a 2 mL auto-sampler vial.

The molecular weight distributions of the cellulose tricarbanilate samples were then analyzed on an Agilent GPC SECurity 1200 system equipped with four Waters Styragel columns (H1, H2, H4, H6), Agilent refractive index (RI) detector and Agilent UV detector (270 nm) using THF as the mobile phase (1.0 mL/min) with injection volumes of 20 μL. A calibration curve was constructed based on eight narrow polystyrene standards ranging in molecular weight from 1.5 × 10^3^ to 3.6 × 10^6^ g/mol. Data collection and processing were performed using Polymer Standards Service WinGPC Unity software (Build 6807). Molecular weights (M_n_ and M_w_) were calculated by the software relative to the polystyrene calibration curve. Number-average degree of polymerization (DP_n_) and weight-average degree of polymerization (DP_w_) were obtained by dividing M_n_ and M_w_ by 519 g/mol, the molecular weight of the tricarbanilated cellulose repeat unit. Polydispersity index (PDI) was calculated by dividing M_w_ by M_n_. 

### 3.6. Cellulose Isolation for NMR Analysis

Isolated cellulose was prepared from the holocellulose sample (1.00 g) by hydrolysis for 4 h in HCl (43.0 mL of 2.5 M) at 43 °C under atmospheric conditions. The isolated cellulose samples were then collected by filtration and rinsed with an excess of DI filtered water, and dried in the fume hood. This procedure is described as hemicellulose removal by acid hydrolysis or the resulting samples as hemi-removed (Hemi) thoughout this paper. The cellulose after isolation was never dried and after their initial analysis stored in a freezer at −4.0 °C to maintain a moisture content greater than 30%.

### 3.7. NMR Analysis

The NMR samples were prepared with moist isolated cellulose (60–30% water content) packed into 4-mm cylindrical ceramic MAS rotors. Solid-state NMR measurements were carried out on a Bruker DSX-400 spectrometer operating at frequencies of 43.55 MHz for ^13^C in a Bruker double-resonance MAS probehead at spinning speeds of 10 kHz. CP/MAS experiments utilized a 5 μs (90°) proton pulse, 1.5 ms contact pulse, 4 s recycle delay and 4–8 K scans. Line-fitting analyses of the spectra were performed using NUTS NMR Data Processing software (Acorn NMR, Inc., Livermore, CA, USA) [58]. A representative statistical average and standard deviation for the various % crystallinity values was generated from isolated cellulose over 5 different trials from untreated *Populus*, which was measured for % crystallinity values by NMR after extraction, holocellulose pulping and HCl treatment. The standard deviation for the % crystallinity values was ~0.6%, and therefore we report a typical error of ~±2%. A similar error analysis was used to determine the typical error associated with % cellulose I_α_, I_β_ and *para*-crystalline cellulose along with cellulose fibril dimensions in Table 1. 

## 4. Conclusions 

The removal of lignin by holocellulose pulping and hemicellulose removal by acid hydrolysis was shown to have little effect on the crystalline ultrastructural components of cellulose based on NMR and GPC results of microcrystalline cellulose. The results of the same experiments on cotton suggest there are some significant effects on amorphous cellulose forming spatially localized acid hydrolysis during lignin and hemicellulose removal. This spatially localized degradation manifests as a change in both cellulose DP and crystallinity. However, based on previous data collected by Hubbell *et al*. [53] and the time-resolved study of the hemicellulose removal on holocellulose pulped *Populus,* these effects seem to be mitigated by the presence of lignin and hemicellulose. 

The results of this study also suggest the traditional method of acid catalyzed removal of hemicelluloses is the most effective, though the corresponding degradation in cellulose DP can be significant as seen in Figure 5 and Figure 6. The data suggests this degradation is invariably accompanied by changes in the crystallinity of cellulose as seen in Figure 4. Although, when limited to nominal cellulosic degradation, approximately less than two cellulose chain breaks, the increase in CrI was consistently ~10% or less. In some cases where the biomass was uncharacteristically low in hemicellulose or crystalline cellulose contents, empirically reducing the acid hydrolysis times can be very important in obtaining accurate degree of crystallinity and ultrastructural information. However, based on the conditions studied for *Populus* pulps, MCC and cotton in this report, accurate NMR cellulose spectra, values of cellulose crystallinity and crystalline allomorphs can be ascertained when holocellulose pulps are treated with 2.5 M HCl at 43 °C for residence times between 1.5 and 4 h. 

## Supplementary Files

Supplementary File 1
