# Supplementary material for: Cellulose Isolation Methodology for NMR Analysis of Cellulose Ultrastructure
Source: Materials (Basel). 2011 Nov 7;4(11):1985–2002. doi: 10.3390/ma4111985 (PMC5448851; doi:10.3390/ma4111985)
Supplement: Supplementary File 1 [file materials-04-01985-s001.pdf]

Supplementary Information

Table S1. Non-linear line-fit parameters of the ^{13}C CP/MAS spectra of untreated microcrystalline cellulose (MCC).

Assignment	^{13}C Chemical Shift	% Area	FWHM (Hz)
I_{α}	89.4	7.9	111
$I_{\alpha+\beta}$	88.7	10.6	100
Para-cystalline	88.2	35.3	170
I_{β}	87.4	8.2	123

Table S2. Non-linear line-fit parameters of the ^{13}C CP/MAS spectra of untreated cellulose from cotton swaps.

Assignment	^{13}C Chemical Shift	% Area	FWHM (Hz)
I_{α}	90.5	9.0	205
$I_{\alpha+\beta}$	89.6	9.2	181
Para-cystalline	88.5	34.5	250
I_{β}	87.7	9.3	235
